# Legius Syndrome in Fourteen Families

**DOI:** 10.1002/humu.21404

**Published:** 2011-01

**Authors:** Ellen Denayer, Magdalena Chmara, Hilde Brems, Anneke Maat Kievit, Yolande van Bever, Ans MW Van den Ouweland, Rick Van Minkelen, Arja de Goede-Bolder, Rianne Oostenbrink, Phillis Lakeman, Eline Beert, Takuma Ishizaki, Tomoaki Mori, Kathelijn Keymolen, Jenneke Van den Ende, Elisabeth Mangold, Sirkku Peltonen, Glen Brice, Julia Rankin, Karin Y Van Spaendonck-Zwarts, Akihiko Yoshimura, Eric Legius

**Affiliations:** 1 Department of Human Genetics, Catholic University of LeuvenLeuven, Belgium; 2 Department of Biology and Genetics, Medical University of GdanskGdansk, Polen; 3 Department of Clinical Genetics, Erasmus Medical CentreRotterdam, The Netherlands; 4 Department of Pediatrics, Erasmus Medical CentreRotterdam, The Netherlands; 5 VU University Medical Center, Department of clinical geneticsAmsterdam, The Netherlands; 6 Department of Microbiology and Immunology, Keio University School of MedicineTokyo, Japan; and Japan Science and Technology Agency (JST)CREST, Chiyoda-ku, Tokyo, Japan; 7 Medische Genetica UZ Brussel, Vrije Universiteit BrusselBrussel, Belgium; 8 Department of Medical Genetics, University of Antwerp and Antwerp University HospitalAntwerp, Belgium; 9 Institute of Human Genetics, University of BonnBonn, Germany; 10 Department of Dermatology, University of Turku and Turku University HospitalTurku, Finland; 11 South West Thames Regional Genetics Unit, St George's University of LondonCranmer Terrace, London, UK; 12 Department of Clinical Genetics, Royal Devon and Exeter HospitalExeter, UK; 13 Department of Genetics, University Medical Center Groningen, University of GroningenGroningen, The Netherlands

**Keywords:** Legius syndrome, *SPRED1*, *NF1*, RAS-MAPK pathway, polydactyly

## Abstract

Legius syndrome presents as an autosomal dominant condition characterized by café-au-lait macules with or without freckling and sometimes a Noonan-like appearance and/or learning difficulties. It is caused by germline loss-of-function *SPRED1* mutations and is a member of the RAS-MAPK pathway syndromes. Most mutations result in a truncated protein and only a few inactivating missense mutations have been reported. Since only a limited number of patients has been reported up until now, the full clinical and mutational spectrum is still unknown. We report mutation data and clinical details in fourteen new families with Legius syndrome. Six novel germline mutations are described. The Trp31Cys mutation is a new pathogenic *SPRED1* missense mutation. Clinical details in the 14 families confirmed the absence of neurofibromas, and Lisch nodules, and the absence of a high prevalence of central nervous system tumors. We report white matter T2 hyperintensities on brain MRI scans in 2 patients and a potential association between postaxial polydactyly and Legius syndrome. © 2010 Wiley-Liss, Inc.

## INTRODUCTION

Legius syndrome (MIM# 611431) was recently identified as a Neurofibromatosis type 1 (NF1)- like syndrome caused by heterozygous germline loss-of-function *SPRED1* (MIM# 609291) mutations (Brems et al., 2007). In the original report five families with an autosomal dominant inherited condition presenting with multiple café-au-lait macules (CALM), axillary freckling, macrocephaly and at times a Noonan-like facial appearance were described. Learning difficulties and/or attention deficit were observed in several children and multiple lipomas were present in several adults. Some typical NF1 (MIM# 162200) features such as Lisch nodules of the iris, neurofibromas and central nervous system tumors were systematically absent.

Pasmant et al. identified five probands with a *SPRED1* mutation in 61 index cases. They confirmed a high prevalence of CALM, freckling and learning disability as well as absence of neurofibromas and Lisch nodules (Pasmant et al., 2009b). Noonan-like dysmorphism was not observed and macrocephaly was reported in only one individual. Lipomas were seen in one family. Spurlock et al. identified 6 probands with *SPRED1* mutations in 85 unrelated patients referred for *NF1* gene (MIM# 613113) testing who were *NF1* mutation negative and had no cutaneous neurofibromas (Spurlock et al., 2009). All affected probands but 1 satisfied NF1 diagnostic criteria. They all had CALM. None had Noonan-like features, macrocephaly, Lisch nodules or cutaneous neurofibromas. No significant developmental or learning problems were observed.

Messiaen et al. performed a genotype-phenotype study in 22 unrelated probands carrying a *SPRED1* mutation identified through clinical testing (Messiaen et al., 2009). Fifty percent of *SPRED1* positive individuals fulfilled NF1 NIH diagnostic criteria based on the presence of more than 5 CALM with or without freckling or a NF1 family history. Symptomatic optic pathway gliomas, neurofibromas or typical NF1 osseous lesions were absent in the affected individuals and there was no increased prevalence of lipomas. Relative macrocephaly was documented in 27% of individuals and language/speech difficulties in 25% of children. In a second cross-sectional study Messiaen et al. performed *SPRED1* mutation analysis in 1318 unrelated patients presenting with a broad range of signs typical for NF1 but no detectable *NF1* mutation (Messiaen et al., 2009). They identified 26 pathogenic *SPRED1* mutations in 33 probands and 7 probable non-pathogenic missense mutations in 9 probands. A *SPRED1* mutation detection rate of 19% was found in families with an autosomal dominant phenotype of CALM with or without freckling and no other NF1 features. Muram-Zborovski et al. sequenced the *SPRED1* gene in 151 individuals with a clinical diagnosis of NF1 and identified 2 *SPRED1*-positive individuals. They both had multiple CALM, intertriginous freckling, learning difficulties and absence of neurofibromas (Muram-Zborovski et al., 2010).

The *SPRED1* gene is a relatively small gene (7 coding exons) located on chromosome 15q14. Most germline mutations truncate the protein, but two pathogenic missense mutations and one in-frame deletion have been reported. Similar to neurofibromin, the protein product of *NF1*, SPRED1 is a negative regulator of RAS-MAPK signalling. Whereas neurofibromin acts as a GTP-ase activating protein that accelerates conversion of active GTP-bound RAS to inactive GDP-bound RAS, SPRED1 is believed to act at the level of RAS-RAF interaction (Wakioka et al., 2001). Both syndromes thus belong to the group of RAS-MAPK pathway disorders or neuro-cardio-facial-cutaneous (NCFC) syndromes (Bentires-Alj et al., 2006; Denayer et al., 2008). Different degrees of cognitive impairment and tumor predisposition are associated with these syndromes. In Legius syndrome one occurrence each of non-small cell lung cancer, Wilms tumor, tubular colon adenoma, (Brems et al., 2007) acute myeloblastic anemia,(Pasmant et al., 2009a; Pasmant et al., 2009b) tenosynovial giant cell tumor, breast cancer and dermoid tumor of the ovary (Messiaen et al., 2009) has been reported. No causal relationship with the germline *SPRED1* mutation has been proven in any of these cases. Whether Legius syndrome is associated with an increased risk for a specific range of malignancies remains unknown. Messiaen et al. estimated that a study of 250 adult patients would be needed to detect rare complications with a prevalence of only 1% (Messiaen et al., 2009). Therefore it remains important to report on new cases of Legius syndrome.

In this study the clinical data of fourteen new probands with Legius syndrome and their affected relatives are described. One missense mutation was characterized functionally.

## MATERIALS AND METHODS

### Study population

After publication of the first report, *SPRED1* mutation analysis was performed in the Department of Human Genetics, Catholic University of Leuven, Belgium in 35 probands with a NF1-like syndrome. DNA samples included patients followed in the Leuven neurofibromatosis clinic as well as samples sent by clinical geneticists from other centres in Belgium or from abroad. Clinical data of 6 affected probands and their relatives carrying a mutation were collected by an extended phenotypical checklist (families 6, 7, 10, 11, 12, 14). In addition clinical data of eight other *SPRED1* -mutation positive families were obtained. Mutation analysis for these cases was performed in the Department of Clinical Genetics, Erasmus Medical Centre, Rotterdam, The Netherlands (families 1, 2, 3, 5, 8, 9, 13) and in the Medical Genetics Centre, Munich, Germany (family 4). Most of these cases showed a phenotype compatible with Legius syndrome: presence of CALM and freckling and absence of neurofibromas and several had been tested before for *NF1* mutations.

Brain imaging data were available for 10 patients (9 MRI, 1 CT).

Height and head circumference at a given age were converted to standard deviations using the growth charts of Flanders 2004 (http://www.vub.ac.be/groeicurven) and Roelants et al., 2009. An individual was recorded as macrocephalic when head circumference was 2 standard deviations above the mean. An individual was recorded to have relative macrocephaly when head circumference was above 2 standard deviations at the age when height would have been at the mean.

### Mutation analysis

Mutation analysis was performed as reported before (Brems et al., 2007). Mutation numbering was based on the cDNA sequence with +1 corresponding to the A of the ATG translation initiation codon in the reference sequence (GenBank accession code: NM_152594.2). For protein numbering the initiation codon is codon 1 (NP_689807.1). Names of all variants were checked using the Mutalyzer program (http://www.LOVD.nl/mutalyzer/).

Amino acid conservation for a novel missense mutation was evaluated with the Conseq program (http://conseq.tau.ac.il/). Bio-informatical prediction of the possible impact of the missense change on the SPRED1 protein was performed with Polyphen (http://genetics.bwh.harvard.edu/pph/).

Mutation analysis in DNA extracted from paraffin-embedded tissue from a vestibular schwannoma was performed with a different set of primers resulting in smaller amplicons.

### Functional analyses

Wild-type *SPRED1* cDNA was cloned into a pcDNA3.1 (Invitrogen) construct encoding an N-terminal Flag-tag. *SPRED1* mutant p.Trp31Cys was generated by PCR-directed mutagenesis and verified by sequencing. Wild-type and mutant *SPRED1* constructs were subcloned in a pmax vector (Amaxa Biosystems). The GAL4/Elk-1 reporter assay was performed as described before (Messiaen et al., 2009). The Elk-1 transcription factor is a substrate for phosphorylation by the activated MAPK pathway. In the GAL4/Elk-1 reporter assay luciferase activity is measured in HEK293T cells co-transfected with pFA-Elk1, pFR-Luc, β-galactosidase and Flag-tagged *SPRED1* constructs after stimulation with epidermal growth factor (EGF).

## RESULTS

### Mutation analysis

Twelve different truncating *SPRED1* mutations were found in 13 unrelated probands: 7 frameshift and 5 nonsense mutations, 11/13 cases were familial. A missense mutation was found in proband 3 and occurred *de novo*. Mutation analysis of DNA extracted from peripheral blood of both parents of proband 3 showed only wild type *SPRED1* sequence. An overview of the different mutations is given in Table 1. Six mutations had not been reported as germline mutations in the literature before (c.93G>T, p.Trp31Cys; c.304dupA, p.Thr102AsnfsX7; c.360dupA, p.Glu121ArgfsX13; c.576_580dup, p.Gln194ProfsX4; c.940C>T, p.Gln314X; c.1045_1046del, p.Arg349GlyfsX4). Trp31 is a highly conserved amino acid residue (Conseq score 7, conserved in Bos Taurus, Mus musculus, Gallus gallus, Xenopus tropicalis, Danio rerio, Drosophila melanogaster). Bio-informatic analysis using Polyphen software predicts the p.Trp3 1Cys mutation to be probably damaging. It may disrupt the function of the EVH1 domain. In the Elk-1 reporter assay overexpression of wild-type *SPRED1* efficiently inhibited the activation of the MAPK pathway after stimulation of the cells with EGF resulting in low luciferase activity. The

**Table 1:** *SPRED1* mutations in fourteen families with Legius syndrome

Family	Exon	Nucleotide change	Amino acid change	Inheritance
1	3	c.52C>T	p.Arg18X	sporadic^b^
2	3	c.70C>T	p.Arg24X	familial
3	3	**c.93G>T**	**p.Trp31Cys**	de novo
4	*J*	c.190C>T	p.Arg64X	familial
5	*J*	c.190C>T	p.Arg64X	sporadic^b^
6	4	**c.304dupA^a^**	**p.Thr102AsnfsX7**	familial
7	4	c.326_329dup	p.Arg110SerfsX2	familial
8	4	c.349C>T	p.Arg117X	familial
9	4	**c.360dupA**	**p.Glu121ArgfsX13**	familial
10	6	**c.576_580dup**	**p.Gln194ProfsX4**	familial
11	8	**c.940C>T**	**p.Gln314X**	familial^b^
12	8	**c.1045_1046del**	**p.Arg349GlyfsX11**	familial
13	8	c.1048_1060del	p.Gly350MetfsX52	familial
14	8	c.1149_1152del	p.Gly385IlefsX20	familial

Mutation numbering was based on the cDNA sequence with +1 corresponding to the A of the ATG translation initiation codon in the reference sequence (GenBank accession code: NM_152594.2). For protein numbering the initiation codon is codon 1 (NP_689807.1).

Bold: novel mutations

a This mutation has already been reported as a somatic mutation in melanocytes of a patient with Legius syndrome

b no DNA from family members available to prove sporadic or familial occurrence of the mutation

**Figure 1:**
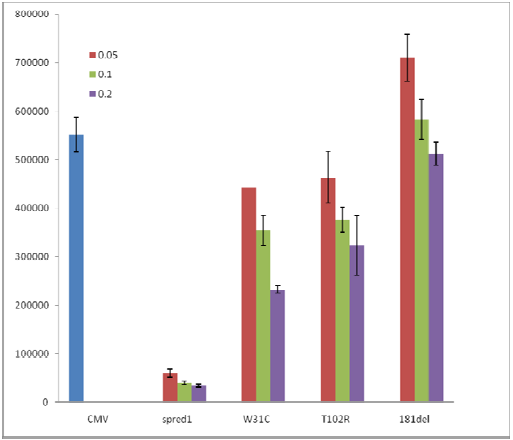
Elk-1 quantitative luciferase assay for Erk activation. Empty vector or vector carrying wild-type (WT; as a control) or mutant SPRED1 (Trp31Cys, W31C; Thr102Arg, T102R; Ile81del, I81 del) were transfected into HEK293T cells along with Elk-1 reporter plasmids. Elk-1 activation is measured as an increase in luciferase activity after stimulation with EGF. Three different amounts of plasmids were transfected: 0.05μg, 0.1 μg and 0.2 μg. The bars represent the average increase in luciferase activity of 3 replicates and error bars represent the standard deviation. Wild-type SPRED1 vector efficiently suppresses EGF induced Elk-1 activation in comparison to empty vector. Similar to the known pathogenic Thr102Arg and Ile81del mutations, Trp31Cys is unable to downregulate the increase in luciferase activity after EGF stimulation.

Trp31Cys SPRED1 mutant was unable to suppress the EGF induced Elk-1 dependent transcription activation confirming that this is an inactivating and therefore pathogenic mutation. (Figure 1)

### Phenotype in patients with germline *SPRED1* mutations

**Figure 2:**
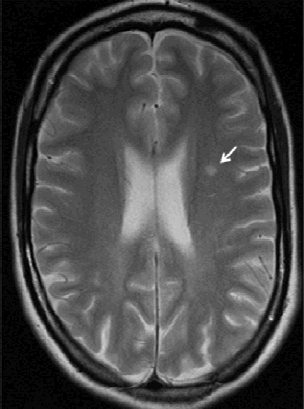
T2 weighed sagittal secion of brain MRI of patient I1 family 11: T2 hyperintense spot is present in the frontal white matter (arrow).

Clinical information was obtained for 14 probands and 16 affected relatives. Phenotypic features are summarized in Table 2. All individuals had more than 5 CALM, except for two adults from the same family who had none. Freckling was present in 10/27 individuals, macrocephaly in 1/24, relative macrocephaly in 2/24. In 20/24 individuals the head circumference was on a higher percentile than the height. Lisch nodules were systematically absent in those patients who had been investigated ophthalmologically (17/30). Neurofibromas were not observed, lipomas were present in 2/15 adults. Information regarding psychomotor development was only available for 17 patients. Five children had motor delay, 5 children delayed speech. Learning difficulties were mentioned in 14/25 individuals. Three individuals were diagnosed with ADHD; in a fourth individual hyperactivity and attention deficit were noticed, but there was no formal diagnosis of ADHD; one patient had attention deficit without hyperactivity (ADD). In another child concentration problems were noted. In 7 patients the diagnosis of Noonan syndrome was considered previously and in 20 patients the diagnosis of NF1 was suspected. Sixteen patients fulfil the NF1 NIH diagnostic criteria based on the presence of more than 5 CALM with or without freckling and a positive family history. One patient had a desmoid tumor of the abdominal wall. A vestibular schwannoma had developed after the age of 50 in patient I1 of family 11. She was said to have the same pigmentary findings as her daughter and granddaughter, but had not been examined clinically. *SPRED1* mutation analysis was conducted on paraffin-embedded tissue from this vestibular schwannoma. In this tissue the familial *SPRED1* germline mutation was confirmed, but no somatic second hit was found in *SPRED1*. T2 hyperintense spots in the subcortical white matter were observed on brain MRI in 2 patients. (Figure 2) Other findings were epilepsy and Parkinsons disease (n=1), unilateral postaxial hexadactyly (n=2), Chiari malformation with syringomyelia (n=1), scoliosis (n=4) with block vertebra (n=1), sensorineural hearing loss (n=1), asymptomatic arachnoid cyst detected by brain MRI (n=1), congenital hypothyroidism (n=1). One patient had a clinical diagnosis of Marfan syndrome, which was confirmed by a pathogenic mutation in the *FBN1* gene (c.3757C>T, p.Gln1253X).

**Table 2:** Clinical features in 14 families with Legius syndrome

Patient	Age (y)/gender	CALM	Freckling	Height:SD	Head circumference: SD	Dysmorphic features	Psychomotor development	Learning difficulties	Behavioral problems	Type of education	Other diagnosis considered	Other
**Family 1**												
**I1**	12/F	>5	inguinal	+0.65	+0.76	Hypertelorism	Normal	Dyslexia, mathematics	No	Special	NF1	Congenital hypothyroidism
**Family 2**												
**I1**	51/M	>5	None	−2.16	+1.94	None	U	No	Rigid, sad, drowsy as consequence of Parkinsonism	Normal	No	Generalized epilepsy since age 39y, Parkinson disease since age 45y, MRI: lesions nucleus caudatus, relative macrocephaly, Surinamese-Indian origin
***II1***	18/M	>5	Axillary	−1.76	+1.33	Low posterior hairline	Normal	Speech problems	Concentration problems and hyperactive, no diagnosis of ADHD	Normal	NF1	Relative macrocephaly, Surinamese-Indian origin
**Family 3**												
***I1***	15/M	>5	None	+0.62	+1.55	Hypertelorism, downslanting palpebral fissures, ptosis, low implanted posteriorly rotated ears, low posterior hairline, mild pterygium colli, pectus carinatum/excavatum	Motor and language delay	Yes	ADHD	Special	NF1	Eczema first year, asthma, strabismus
**Family 4**												
***I1***	36/F	>5	None	−1.18	+0.94	None	U	No	No	Normal	No	5 spontaneous abortions, 1 stillbirth at 6m pregnancy, unilateral postaxial polydactyly foot
***II1***	3/M	>5	None	−2.57	−0.97	Downslanting palpebral fissures, ptosis, low implanted posteriorly rotated ears, widely spaced nipples	Considerable psychomotor delay in all fields, especially speech	/	Attention deficit, no hyperactivity noted	Special kinder-garten	NF1, Noonan	Born preterm (32w) due to IUGR and placental insufficiency, unilateral postaxial polydactyly hand without bony structures
**Family 5**												
***I1***	11/M	>5	Axillary, inguinal	−2.33	+0.54	Hypertelorism, pectus excavatum	Delayed motor milestones	Non-verbal learning disorder	No	Normal	NF1, Noonan	
**Family 6**												
***I1***	40/F	>5	U	U	U	U	U	Yes	U	Normal	No	Desmoid tumor abdominal wall
***II1***	1/F	13	U	U	U	U	U	/	U	/	NF1	Dizygotic twin
**Family 7**												
***I1***	58/F	None	None	−2.27	+0.65	Low	U	U	U	U	No	Hysterectomy implanted ears, proöptosis, coarse facial features
**II1**	37/F	None	None	+0.27	+1.35	None	Normal	Yes, mathematics	U	U	No	Obesity
***III1***	11/F	>5	Axillary	−1	+0,06	Pectus excavatum	Normal	Yes, mathematics	ADHD	Normal, FSIQ: 85, VIQ: 93, PIQ: 80	NF1, Noonan	Placental insufficiency, oligohydramnios, dysmaturity, transient trombopenia neonatally, Brain MRI: bilateral temporal subcortical white matter lesions (T2 hyperintense)
**Family 8**												
***III1***	39/F	>5	None	+0.09	−0.12	Mild synophris	Normal	No	No	Normal	NF1	Migraine, some skintags in neck and face
III6*	36/M	>5	None	+1.2	+0.17	Downslanting palpebral fissures, higharched palate, protruding ears	Normal	No	No	Normal	NF1	Motoric clumsiness, mild lumbal scoliosis, varices L lower leg
**IV1**	13/M	>5	None	−0.75	+1.12	Telecanthus, low implanted ears with thick helix	Normal	No	No	Normal	NF1, Noonan	Immature motor skills, physiologic tremor
***IV3***	9/M	>5	None	−0.45	+0.25	Hypertelorism, low implanted, posteriorly rotated ears with thick helix, short neck, mild webbing	Normal	Yes, mathematics, one doublure	No	Normal	NF1, Noonan	Immature motor skills
**Family 9**												
***II1***	6/M	>5	None	+3	+0.19	Epicanthal folds, ptosis, pectus carinatum	Normal	Yes	No	Special	NF1	Marfan syndrome caused by pathogenic *FBN1* mutation (c.3757C>T, p.Gln1253X), prolapse of mitral and tricuspid valve, wide aortic root
**Family 10**												
**II1**	19/M	several	None	−0.23	−1.21	Maxillary and malar hypoplasia, prognathism, downslanting palpebral fissures	Walked at 18m, required speech therapy	Mild	No	Normal (university)	No	
**II2**	15/M	>5	Minimal axillary	−0.77	+0.6	Maxillary and malar hypoplasia, prognathism, Noonan facies, downslanting palpebral fissures, pectus excavatum	General delay	Mild	No	Normal	NF1, TWIST, FGFR3	Chiari 1 malformation with syringomyelia with secondary scoliosis, normal echocardiogram
**II5**	12/M	Many	None	−2	−0.07	Malar hypoplasia, prognathism, low set ears, downslanting palpebral fissures, high narrow palate	U	Mild	No	Normal	No	
**II6**	10/F	Many	None	−1.47	−0.33	Malar hypoplasia, prognathism, long narrow face	Speech therapy	Mild	No	Normal	No	Thoracic scoliosis requiring surgery
**Family 11**												
***I1***	39/F	>5	Axillary, inguinal	U	+2.41	Hypertelorism, broad nasal bridge, small nose tip, large mouth with thick underlip, upslanting palpebral fissures	U	Concentration problems	No	Normal	NF1	Congenital scoliosis, block vertebra L4-L5, sensorineural hearing loss, frequent headaches, Brain MRI: white matter lesions frontal and temporo-occipital (T2 hyperintense)
**Family 12**												
***I1***	38/M	>5	General	−2.32	+0.28	No	U	No	No	Normal	No	
***II1***	12/M	>5	None	+0.19	+0.44	No	Normal	No	Anxiety, suspicion of autism	Normal	NF1	
**II2**	8/M	>5	Inguinal	+0.4	+1	No	Normal	No	No	Normal	NF1	
**Family 13**												
**I1**	48/M	>5	None	−2	−1.5	Hypertelorism, ptosis, low implanted posteriorly rotated ears, widely spaced nipples, pectus excavatum	U	U	U	U	NF1, Noonan	lipomas
**II1**	15/M	>5	None	−2.71	+0.44	Hypertelorism, low implanted posteriorly rotated ears, pectus excavatum	U	No	ADHD	Normal	NF1, Noonan	Laryngomalacia, cubitus valgus, lipomas
**Family 14**												
I1*	74/F	>5?	U	U	U	U	U	U	U	U	U	Unilateral vestibular schwannoma after age 50y
***II1***	48/F	>5	Axillary, inguinal	−1.18	U	No	U	No	No	Normal	NF1	Brain MRI: asymptomatic arachnoid cyst right temporal lobe
**III1**	20/F	>5	Axillary, inguinal	−0.27	U	No	Normal	No	No	Normal	NF1	

SD: standard deviation; U: unknown; L: left; R: right; MRI: magnetic resonance imaging; ADHD: attention deficit hyperactivity disorder; FSIQ: full-scale intelligence quotient; VIQ: verbal intelligence quotient; PIQ: performance intelligence quotient; IUGR: intrauterine growth retardation

* clinical phenotype present, but no mutation analysis performed in this patient

## DISCUSSION

We report on the second largest series of families with Legius syndrome. In this report we describe 30 new individuals from 14 families. Our results confirm previous findings that Legius syndrome is characterized by the presence of CALM with or without freckling and the absence of typical NF1 complications such as Lisch nodules and neurofibromas. In a subset of patients we find Noonan-like facial features (n=12) and/or sternal abnormalities (n=7; one of these patients had an additional diagnosis of Marfan syndrome, which is also associated with sternal abnormalities). No congenital vascular malformations were present in this cohort. One vestibular schwannoma and one benign tumor of the abdominal wall (desmoid tumor) were observed. Both tumor types had not been reported before in association with Legius syndrome and it is unclear whether there is a causal relationship between these tumors and the germline *SPRED1* mutation. Learning difficulties were mentioned in 14/25 patients (56%), three patients were diagnosed with ADHD and one with ADD. Combining the data in the literature with that of the current report (142 individuals) learning difficulties were observed in 26 individuals (18.3%) with Legius syndrome, delayed psychomotor development (mostly confined to specific speech delay) in 13 (9.2%), and hyperactivity, attention problems or ADHD in 14 (9.9%). Whether Legius syndrome is associated with a specific neurocognitive profile warrants further investigation.

Focal areas of high signal intensity on T2-weighed or FLAIR images of brain MRI, also denoted UBOs (Unidentified Bright Objects), are found in about 70% of children with NF1 (Gill et al., 2006; North, 2000). UBOs occur most often in the basal ganglia, cerebellum, thalamus, brainstem and subcortical white matter. They are not associated with focal neurological deficits, but the pathological correlate remains controversial (North, 2000). The number and intensity of UBOs diminish with age in the basal ganglia, cerebellum, brainstem and thalamus, whereas lesions in the cerebral hemispheres and hippocampus do not change in prevalence over time, suggesting a different pathologic background (Gill et al., 2006). There is no consensus on the relationship between UBOs and cognitive impairment in NF1. We report two patients (one child and one 39 years old woman) with Legius syndrome and aspecific T2 hyperintense lesions on brain MRI. Thus the presence of such a lesion cannot be used to exclude the diagnosis of Legius syndrome. However at an adult age these lesions are aspecific and not uncommon in the general population. To gain better insight into the prevalence of these and other structural brain lesions in patients with Legius syndrome it would be interesting to systematically perform brain MRI in all patients in whom the diagnosis is made.

Unilateral postaxial polydactyly has been observed both by Messiaen et al. (in one case) and in this report (two cases) to be associated with Legius syndrome. Combining the data in the literature with that of the current report clinical details of 142 individuals with a *SPRED1* mutation have been described (Brems et al., 2007; Messiaen et al., 2009; Muram-Zborovski et al., 2010; Pasmant et al., 2009b; Spurlock et al., 2009). According to the EUROCAT data the prevalence of any type of polydactyly at birth in the European member states was 8.11 cases per 10000 births in the 2000-2007 period. The highest prevalence in a specific region of the European Union in that time frame was 15 cases per 10000 births (Strasbourg, France). Comparing the prevalence of polydactyly in Legius syndrome (3/142) with the maximal birth prevalence in the European Union (15/10000) results in a significant difference (p= .0014 for 3 or more cases in a group of 142, binomial distribution). It is interesting to note that a similar figure was reported in an Italian study in children with NF1. Ruggieri et al. reported polydactyly in 4 children in a total group of 135 children with NF1 (Ruggieri et al., 1999).

Germline *SPRED1* mutations are found in all exons of the gene and although some recurrent mutations have been reported, no clear mutational hotspots are present. The majority of reported *SPRED1* mutations are predicted to result in a premature stop codon (nonsense, frameshift, splice-site, out-of-frame insertions and deletions). One in-frame deletion and 11 different missense mutations have been reported. By means of functional analyses 2 missense mutations were classified as pathogenic and 7 as rare benign variants.(Messiaen et al., 2009). Two missense mutations were not characterized (Pasmant et al, 2009b, Spurlock et al., 2009). We characterized an additional missense mutation and classify this Trp31Cys mutation as pathogenic based on de novo occurrence in the family, amino acid conservation and functional analysis.

Combining the data in this report and previous publications, one can conclude that the highest chances of finding a *SPRED1* mutation are in familial cases of CALM with or without freckling. Sporadic cases of CALM are more likely due to mosaicism for NF1(Kehrer-Sawatzki and Cooper, 2008). Although the diagnosis of NF1 is made clinically based on the NIH criteria, the importance of mutation analysis for genetic counselling is increasing with the discovery of allelic and non-allelic NF1 variants. Especially in young children the diagnosis of NF1 can be difficult since only approximately half of children with NF1 and no known family history meet the NIH criteria by age one year, because many features of NF1 increase in frequency with age. The more general availability of *NF1* mutation analysis allows for more general use of *NF1* mutation testing also in young children. Children with typical multiple CALM who test negative *for NF1* are candidates for *SPRED1* mutation analysis. In sporadic cases the yield will be low (2.4%)(Messiaen et al., 2009). In children testing negative for mutations in both *NF1* and *SPRED1* mosaicism for *NF1* should be suspected (Maertens et al., 2007). As has been shown in this and previous reports Legius syndrome is associated with a milder phenotype than NF1. Likewise a mild NF1 variant associated with a 3-basepair inframe deletion of exon 17 (c.2970-2972 delAAT) has been described in which neurofibromas are rare and multiple CALM may be the only apparent manifestation (Upadhyaya et al., 2007). In contrast, patients with a microdeletion of the *NF1* region tend to have a more severe phenotype associated with large numbers and more early appearance of cutaneous neurofibromas, a higher risk for development of malignant peripheral nerve sheath tumors, more severe cognitive impairment and sometimes somatic overgrowth with large hands and feet and dysmorphic facial features (Mensink et al., 2006).

In conclusion, we report clinical details of 30 probands from 14 families with pathogenic *SPRED1* mutations. Six novel germline mutations were found. The p.Trp31Cys missense mutation is a pathogenic missense mutation. Our results confirm previous reports that Legius syndrome shares clinical features with NF1 but has a milder phenotype, although serious problems can be present such as learning difficulties. Therefore we can recommend less stringent surveillance of these patients than for patients with NF1. We would recommend routine screening for developmental delays and behavioural and learning problems. However physical examination by a clinical geneticist or physician familiar with Legius syndrome every 3 years during childhood seems sufficient. A possible association with polydactyly is suggested by the finding of two cases in this report and one in a previous report.
